# Regional Influenza Prediction with Sampling Twitter Data and PDE Model

**DOI:** 10.3390/ijerph17030678

**Published:** 2020-01-21

**Authors:** Yufang Wang, Kuai Xu, Yun Kang, Haiyan Wang, Feng Wang, Adrian Avram

**Affiliations:** 1 School of Statistics, Tianjin University of Finance and Economics, Tianjin 300222, China; 2 School of Mathematical and Natural Sciences, Arizona State University, Phoenix, AZ 85069, USA; kuai.xu@asu.edu (K.X.); fwang25@asu.edu (F.W.); adiman2370@gmail.com (A.A.); 3 Science and Mathematics Faculty, Arizona State University, Mesa, AZ 85212, USA; yun.kang@asu.edu

**Keywords:** flu prediction, sampling tweets data, PDE model

## Abstract

The large volume of geotagged Twitter streaming data on flu epidemics provides chances for researchers to explore, model, and predict the trends of flu cases in a timely manner. However, the explosive growth of data from social media makes data sampling a natural choice. In this paper, we develop a method for influenza prediction based on the real-time tweet data from social media, and this method ensures real-time prediction and is applicable to sampling data. Specifically, we first simulate the sampling process of flu tweets, and then develop a specific partial differential equation (PDE) model to characterize and predict the aggregated flu tweet volumes. Our PDE model incorporates the effects of flu spreading, flu recovery, and active human interventions for reducing flu. Our extensive simulation results show that this PDE model can almost eliminate the data reduction effects from the sampling process: It requires lesser historical data but achieves stronger prediction results with a relative accuracy of over 90% on the 1% sampling data. Even for the more aggressive data sampling ratios such as 0.1% and 0.01% sampling, our model is still able to achieve relative accuracies of 85% and 83%, respectively. These promising results highlight the ability of our mechanistic PDE model in predicting temporal–spatial patterns of flu trends even in the scenario of small sampling Twitter data.

## 1. Introduction

Accurate and timely flu forecasting is critical for reducing and mitigating flu epidemics during flu seasons. Due to the manual data collection that takes weeks to process [1], conventional influenza surveillance often describes and characterizes the outbreaks of flu that have already happened. The government health agencies regularly report and share official statistics on the flu trend; however, these statistics often fail to shed light on the latest development of flu epidemics due to significant time delay [2]. For example, the Center for Disease Control and Prevention (CDC) in the United States collects influenza surveillance data on a weekly basis starting from Sunday each week and ending on the following Saturday. Typically, each surveillance participant submits the summarized weekly data to CDC by Tuesday afternoon of the following week. Finally, CDC compiles and analyzes the summarized weekly data from distributed regional participants and reports the national influenza surveillance summary each Friday of the following week [3].

There exist many computational approaches to model epidemic spread, ranging from very detailed agent-based approaches [4,5] to structured meta-population models [6,7,8]. Moreover, there also exist publicly available software, for instance “GLEaMviz” [9,10], which simulate the spread of human-to-human infectious diseases across the world. However, all these models are based on real spatiotemporal influenza data about influenza-like illness (ILI) cases. Although these models can reveal the transmission characteristics of the infectious diseases and some other properties, the prediction ability needs to be improved due to a certain time lag from the real flu data.

Our work attempts to apply the timely data from social media to detect the real-time prediction for flu. In recent years, the rapid increase of online social media has contributed tremendous and valuable real-time data on flu. Every day, thousands of people share their flu statuses and recovery process via online social media such as Twitter [1]. To some extent, analyzing the rich data on flu-related tweets potentially reveals the actual flu situations in the real world. Such data, if efficiently studied, will bring new opportunities for timely monitoring flu outbreaks and accurately predicting flu spread.

Recently, the surveillance, monitoring, and analysis of epidemic outbreaks and spread via large-scale social media data have become an important and practical tool for government agencies and public health organizations to control and prevent the flu from spreading [2,11,12,13,14,15,16]. Among these studies, a few efforts focus on flu twitter data itself, for instance for explicitly modeling the distinction between flu awareness and real flu infection [11,12]. This provides a rich set of high-quality datasets for efficient forecasting, while some studies apply a temporal topic model [13,14] or machine learning method [15,17,18,19] to estimate the flu trends. Recently, a few mathematical models such as partial differential equation (PDE)-based approaches are proposed to predict the information diffusion over online social networks [16,20,21]. However, based on the data from social networks, all the existing PDE models referred above are built without consideration human activity.

Unlike such prior research, our paper aims to incorporate human efforts on flu prevention and mitigation into a specific model to quantify these effects based on active human intervention. In reality, humans have been battling the flu especially in the full-swing flu season and the efforts of human interventions have been incorporated into traditional structured meta-population models [6].

In addition, it is necessary to develop robust algorithms that are able to analyze and predict flu tweets in the context of data sampling. When making predictions about influenza based on social media data, the robustness of the model to the data is important. Here robustness means that the model still shows a good prediction ability for influenza situations even based on sampling data. The sheer volume and velocity of online social media data naturally lead to challenges and obstacles in collecting, analyzing, and modeling flu tweets. Therefore, to the researcher and the developer community, sampling is a natural and popular choice for online social media sites to share data. For example, the sampling tweet API (Application Programming Interface) available at the Twitter developer platform returns a random set of tweets in real time. This effectively reduces the data size for storage and computations. However, the sampling process substantially reduces the number of tweets, which in turn creates difficulty in characterizing and predicting flu tweets [20,21].

Overall, the present work aims to explore flu infection prediction based on sampling data of flu tweets volume. Specifically, we first provide a simple yet effective process to emulate the data sampling. Subsequently, we develop a specific PDE model to characterize and predict the spatial– temporal dynamics of flu tweets volume across the 10 geographical regions defined by the Center for Disease Control and Prevention (CDC) [22]. Finally, we systematically validate the prediction ability of the PDE model in the scenario of small sampling Twitter data. Furthermore, we also predict the impact of human control on influenza.

Although PDE models have been extensively used to describe flu trends, most are structured meta-population models based on real data of ILI cases which are very difficult to collect in real time. Therefore, it is crucial to develop a specific PDE model based on data from social media while achieving high predictive ability based on sampling social data. Our experimental evaluations show that our proposed new PDE model has promising prediction ability even under the scenarios of small sampling Twitter data.

## 2. Sampling Data

In this section, we first present the flu tweet datasets used in this study, subsequently we describe our data preprocessing steps, particularly data sampling and normalization processes, for developing new flu prediction models based on sampled flu tweets data from social media. In addition, we also shed light on the potential information loss from the original data in the scenarios of aggressive sampling strategies.

### 2.1. Dataset

In this study, we explore the publicly available Twitter filter streaming APIs to collect complete flu-related tweets in real time [23]. Based on a set of predefined keywords, user identification numbers, and geographical locations, the filter streaming API returns all the matching public tweets. Given that tracking and collecting all the important topics and influential users is impractical and resource intensive, we adopted the strategy of previous studies [2] to carefully select five keywords on influenza epidemiology: flu, influenza, cold, cough, and headache. Throughout this paper, the real-time tweet data streams matching the five keywords and collected via the filter streaming API are referred to as full tweets.

**Figure 1:**
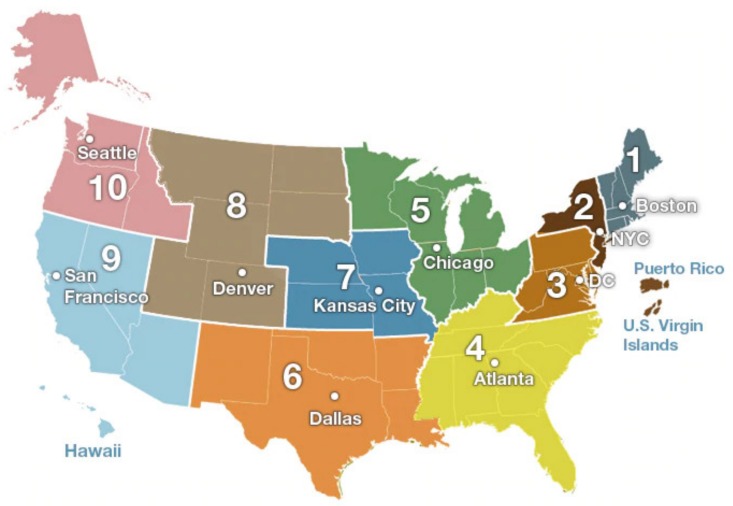
National Center for Chronic Disease Prevention and Health Promotion regions 1–10 represent 10 different CDC regions in the United States.

**Figure 2:**
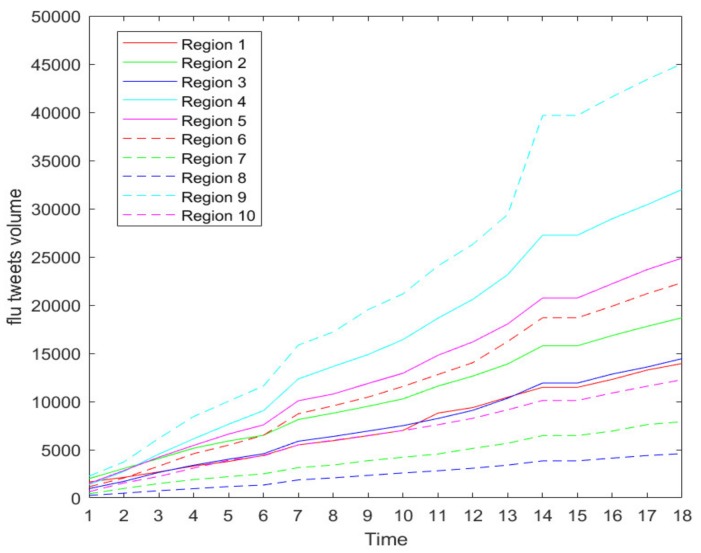
Flu full tweets from the 40th week in 2018 (marked as 1 in the *x*-axis) to the 5th week in 2019 (marked as 18 in the *x*-axis), where lines with different colors represent different Center for Disease Control and Prevention (CDC) regions and flu tweets volume in the *y*-axis represents flu tweet counts on Twitter.

Our twitter data collection of the 10 CDC regions spanned 18 weeks starting from early October in 2018, i.e., the 40th week of 2018, and ending in early February in 2019, i.e., (Supplementary Materials Table S1) the 5th week of 2019. The 10 CDC regions are shown in Figure 1. The duration covers the prophase and metaphase of a flu season. As the CDC reports, seasonal influenza viruses are detected year-round in the United States. The exact time and duration of flu seasons may vary, but the influenza activity often begins to increase in October, peaks between December and February, and lasts as late as May [22]. Figure 2 shows the increasing trend of weekly new flu tweets through all 10 CDC regions during our data collection period. The growing trend confirms that the flu season is in full swing.

### 2.2. Data Preprocessing

Next, we describe our data sampling and normalization processes on the collected flu tweet datasets. These common data preprocessing steps provide validated and appropriate input for our proposed PDE-based prediction models. The sheer data volume and velocity of online social media data has driven sampling to become a natural and popular choice of social media analytics. Meanwhile, as illustrated above, the flu season is in full swing, and thus the total flu tweets volume, i.e., the total flu twitter counts, are growing fast. Therefore, data sampling is a must. To prevent the larger value input attributes from overwhelming smaller value inputs and to effectively decrease prediction errors, we chose to perform data normalization on the sampling data.

**Figure 3:**
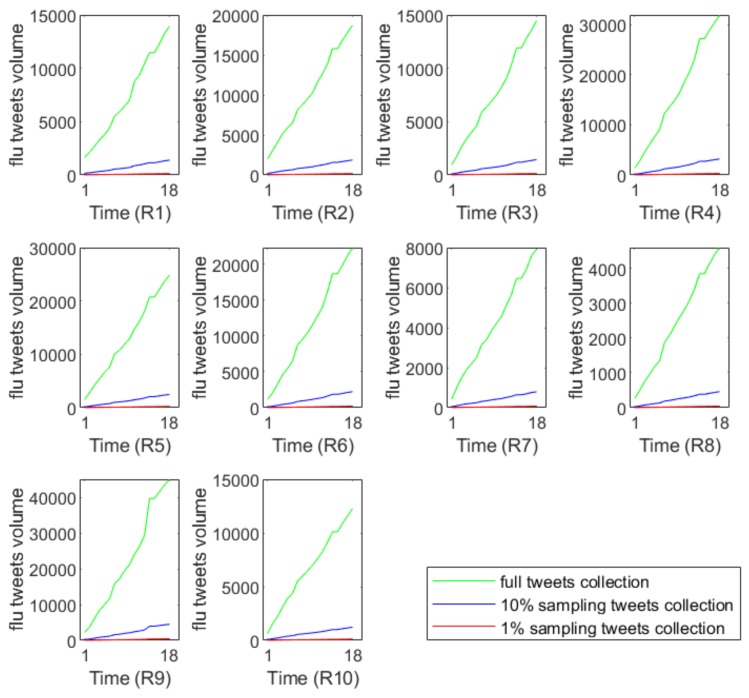
The data collection of flu tweets from random sampling and full tweets. *x*-axis represents the research time period of 18 weeks from the 40th week of 2018 to the 5th week of 2019, which are marked 1–18 in the *x*-axis. Flu tweets volume (flu tweet count) of each CDC region from Region 1 (marked as R1) to Region 10 (marked as R10) is shown in each subfigure.

**Data sampling:** To characterize the impact of sampling on Twitter flu prediction, we simulated a random sampling process via a simple yet widely used sampling strategy to generate a sampled Twitter flu dataset. As illustrated in [20], tweets from Twitter data streams will be selected into the samples with the same probability p during a random sampling process. Thus, the projected sampling flu tweets volume n on a weekly basis can be obtained by n=[N×p], where N is the total number of weekly full tweets on flu. [•] is the integer operation with rule of rounding. For instance, [0.3] equals 0, [5.3] equals 5, and [5.5] equals 6. Intuitively, the effect of random sampling could potentially change the underlying distributions of the flu tweets. Figure 3 demonstrates that the sampling tweets volumes have very different growth trends compared with the full data. This phenomenon reflects the information loss during the simulation process of random sampling.

**Data normalization:** To prevent the larger value input attributes from overwhelming smaller value inputs and to effectively decrease the prediction errors, we first normalized all the known historical sampling values of the tweets volume to a specific range of [0,m] via linear scaling. Here *m* is predefined and in the present study we chose m=5 in order to align with the official flu case levels 1–5 defined by CDC. Specifically, we defined M as the maximum value of tweets volumes, i.e., the maximal flu tweets volume over the entire flu season. We normalized the flu tweet counts by the linear transform $y=\frac{m}{M}x$, where x is the real tweet volume, y is the normalized tweet volume.

During the data sampling and normalization steps, we noticed an interesting observation of the sampling impact on the underlying distributions of the flu tweets in the scenarios of very high sampling ratios. Specifically, supposing $\left\{x\right\}$ is the original dataset of flu tweets volume in each CDC-regions at each observation time. After first data sampling and then data normalization on $\left\{x\right\}$, that is $y=\frac{m}{\left(Mp\right)}[xp]$, we obtain a sampling and normalized dataset $\left\{y\right\}$, which is referred to as the new dataset below. The new datasets $\left\{y\right\}$ are different with and without data sampling, as the former is $y=\frac{m}{\left(Mp\right)}[xp]$ and the latter is $y=\frac{m}{M}x$ and p is the sampling rate and as [xp] may not equal to xp. Especially in the cases of [xp]=0, sampling results in many zeros occurring in the sampling dataset and this obviously results in the loss of the original data in x. Our numerical experiments below have also confirmed that when p is small enough, the prediction results become worse due to the heavier data loss introduced by very high sampling ratios.

## 3. PDE Model

In this section, we introduce a specific PDE model to characterize the spatial–temporal dynamics of flu tweets volume and consider 10 CDC regions of the United States as examples in the model description.

Let F(x,t) represent the count of flu tweets in CDC region x at a given time t. The changing rate of F(x,t) depends on three processes: (i) The movement of people, such as traveling and commuting between regions, that contributes to the spreading of flu [24]; (ii) in each CDC region, people become newly infected or have recovered from the flu [25,26]; (iii) people have taken active efforts to reduce and mitigate flu symptoms [27]. Therefore, the dynamics of flu tweets volume can be captured by Equation (1).

(1) $$\begin{array}{r} \frac{\partial F(x,t)}{\partial t}=\frac{\partial }{\partial x}(d(x)\frac{\partial F(x,t)}{\partial x})+r(t)F(x,t)l(x)-g(F(x,t)),\\ F(x,1)=\psi (x),L_{1}<x<L_{2},\\ \frac{\partial F}{\partial x}(L_{1},t)=\frac{\partial F}{\partial x}(L_{2},t)=0,t>1, \end{array}$$

where the function g(F(x,t)) is the rate of the reduced flu cases due to human efforts. During the period of high morbidity, people are often advised to take effective flu prevention actions to reduce the contact rate, such as wearing masks and staying indoors. We apply the function term g(F(x,t)) to describe the potential reduced flu exposure and infection rate. Specifically, the function g(·) is an increasing and upper-bounded function of F(x,t) with g(F(x,t)) and g(0)=0. In this study, g(F(x,t)) is given as

(2) $$g(F)=\frac{cF}{k+F},$$

where $\frac{F}{k+F}$ is the Michaelis–Menten equation, which approaches 1 as F is large; c>0 represents the maximum reduction rate of flu cases due to human behavior changes or government measures, and its unit is flu tweets counts over time k has the same unit as F and it denotes the twitter flu volume at which the reduction rate is $\frac{1}{2}c$. By adjusting the parameter c, this model allows us to examine the effect of enhancing interventions to control and prevent flu epidemics. Actually, a similar form of g(•) has used to describe human behavior changes such as wearing masks and reducing outdoor behavior to prevent flu epidemics in [27].

The term r(t)F(x,t)l(x) represents the changing rate of the aggregated Twitter flu volume from a local CDC region at location x and time t, and the term has the same unit of F over a unit of time. This factor is widely used to describe the growth of bacteria, tumors, or social information over time [25,26]. Specifically, the function r(t)>0 is the growth rate per capita; its unit is 1/time; it represents the intrinsic growth rate of flu cases at time t for all CDC regions. Function r(t) increases with time t as the flu season starts, and naturally decreases when the flu season starts to end. Therefore, we choose

$$r(t)=b_{1}+e^{-b_{2}{\left(t-a\right)}^{2}}$$

to describe these patterns with parameters $b_{1}>0,b_{2}>0,a>0$, to be determined by the collected flu tweets data.

The location function l(x) describes the spatial heterogeneity of flu and it is a dimensionless factor, which depicts different states in the 10 CDC regions. The function l(x) is built through a cubic spline interpolation [28], which satisfies $l(x_{i})\equiv l_{i},i=1,2,\ldots ,10,$ where $x_{i}$ represents the location of CDC region i. l(x) is determined by the latest Twitter flu data.

The term $\frac{\partial }{\partial x}(d(x)\frac{\partial F(x,t)}{\partial x})$ denotes the regional spreading of flu between different CDC regions, where d(x) has the unit of ${\mathrm{length}}^{2}/\mathrm{time}$ and it measures how fast flu spreads across different CDC regions. In epidemiology [24], the term $\frac{\partial }{\partial x}(d(x)\frac{\partial F(x,t)}{\partial x})$ has been widely used for describing the spatial spread of infectious disease. Here we assume *d*(*x*) to be constant, i.e., d(x)≡d>0. 

The Neumann boundary condition $\frac{\partial F}{\partial x}(L_{1},t)=\frac{\partial F}{\partial x}(L_{2},t)=0,\;t>1$ is applied in this paper [29]. For simplicity, we assume no flux of flu disease spread across the boundaries at $x=L_{1},L_{2}$.

The initial function F(x,1)=ψ(x) describes the early counts of Twitter flu tweets in every CDC region, which can be constructed from the historical data of Twitter flu count by cubic spline interpolation.

Many standard structured meta-population models characterize flu cases by a system of PDEs, describing the dynamics of susceptible, infected, and recovered populations. These models are built based on real ILI data with time delays and thus real-time prediction cannot be guaranteed. Though our previous work [2] applies a PDE model to study regional level influenza with geotagged twitter data, the model is simple without considering the affects from human intervention. The new PDE model in Equation (1) developed in the present work fills this research gap by concentrating on sampling flu tweets data from social media, and incorporating factors from human movement between regions, newly infected or recovered cases in each region, and active human interventions.

## 4. Predictive Modeling

The basic mathematical properties of the proposed PDE model in Equation (1) such as existence and uniqueness can be established from the standard theorems for parabolic PDEs in [30].

Below, we study the robustness of our developed PDE-based prediction model and validate whether the model has similar or acceptable prediction performance with Twitter sampling data as full data. In other words, we are interested in understanding the prediction ability of the PDE-based prediction model under a spectrum of sampling scenarios. The procedure of predictive modeling for the flu tweets is summarized as follow:

**Flu tweet predictions**: In the prediction process of the research time period, the model parameters are time varying but under the same structured PDE. For forecasting the flu tweets of a given day, we first train the parameters of the PDE model and then solve the PDE for prediction. Specifically, weeks 1–3, 2–4, …, 15–17 are used as the training data, and we predict the flu tweets for the following weeks 4, 5, …,18, respectively. In this study we only require the last three weeks of the history data to train the model, which is much less than the historical data expected in [2].

In the process of performing prediction, parameters in the PDE model are real-time determined with history flu tweets volume. Essentially, this is a list of multiparameter inverse problems of parabolic equation. Combing the advantages of local and global methods, in the present work we adopt hybrid methods: First a tensor train global optimization [31] is used to explore the parameter space to locate the starting points, and then Nelder–Mead simplex local optimization method [32] is used to search the local optimization, where the Nelder–Mead simplex method corresponds to the fminsearch function in MATLAB. After determining each of the model parameters, we use the fourth-order Runge–Kutta method to solve the PDE for one-step forward prediction numerically. For one-step forward prediction, we perform 800 loop iterations; the total training and prediction process for the flu tweets count of this prediction needs almost 63 s in the software version of R2018a on a laptop with 8 GB RAM and i7-6500U CPU at 2.50 GHz.

**Reversing the data normalizing and sampling process**: Once we obtain the predicted flu tweets volume via the developed PDE model, we transform the predicted tweets volumes based on sampling data into the full version via reversing the data normalizing and sampling process.

**Measuring the prediction ability of the PDE-based model**: Lastly we compare the predicted flu tweets volumes (that have been transformed through reversing the data normalizing and sampling process) with the observed flu tweets volumes, i.e., the ground truth, to quantify the prediction accuracy of the flu tweet volume. The $\mathrm{relative}\;\mathrm{accuracy}=1-\frac{\left|x_{real}-x_{predict}\right|}{x_{real}}$ is applied to measure the prediction accuracy, where $x_{real}$ is the full flu tweet volume at every data collection time point and $x_{predict}$ is the predicted tweet volume, which has been inverse normalized and inverse sampled.

**Figure 4:**
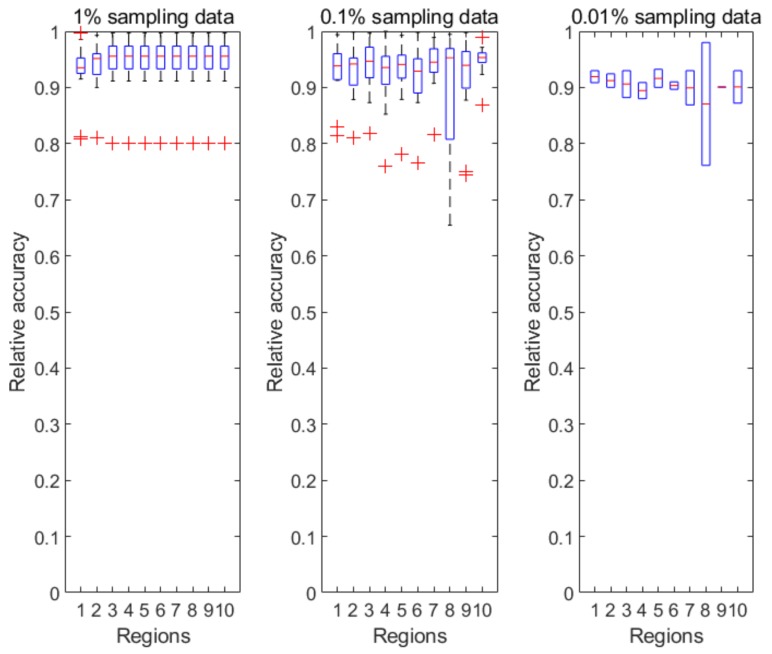
The relative accuracy in each CDC region (regions 1–10) from the 40th week of 2018 to the 5th week of 2019, which covers the prophase and metaphase of a flu season. Here the relative accuracy is the conventional definition as $1-\frac{\left|x_{real}-x_{predict}\right|}{x_{real}}$ where $x_{real}$ is the actual full flu tweet volume at every data collection time point and $x_{predict}$ is the predicted tweet volume, which has been inverse normalized and inverse sampled.

Figure 4 illustrates the prediction accuracy in 10 CDC-defined regions every week from the 40th week of 2018 to the 5th week of 2019. Clearly, the relative accuracy of almost all the regions during the research time period is above 90% based on the 1% sampling data. As the sampling ratio changes from 1% to 0.01%, the accuracy of the predicted results almost unsurprisingly decreases due to the accelerated information loss. However, the PDE-based prediction model is still able to achieve a relative accuracy of 85% or above for all CDC regions.

**Figure 5:**
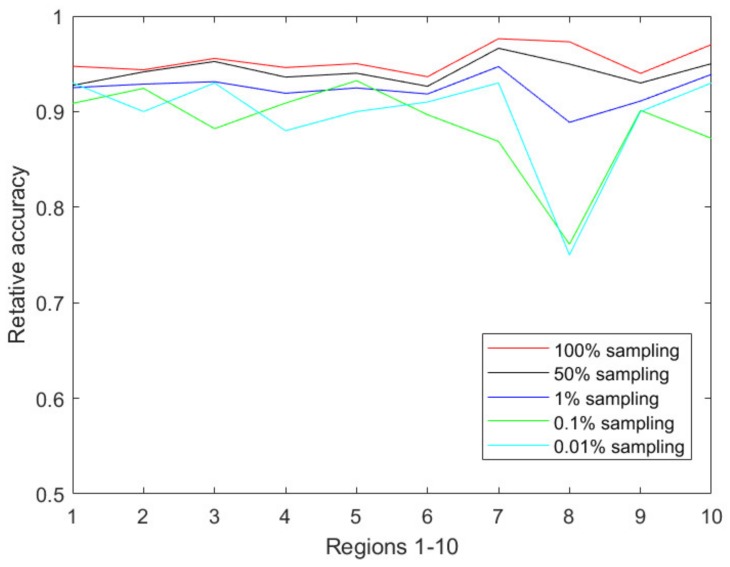
The average relative accuracy of the 10 CDC regions with various sampling ratios during our data collection period. *x*-axis represents the CDC regions from 1 to 10. Lines with different colors represents that the prediction accuracies are based on different sampling data.

Table 1 shows the average relative accuracies of the CDC regions from Region 1 to Region 10, forecasted based on different sampling ratios. As shown in Table 1, our developed PDE-based prediction model has a strong prediction ability even under aggressive sampling scenarios during an active flu season. The prediction accuracy of Region 8 is relatively lower than the others. As shown in Figure 2 of Section 2.1, Region 8 has the lowest tweet count among all the CDC-regions, thus we conjecture that the relatively lower accuracy is due to the smaller size of tweet data on flu. As we continue to increase the sampling ratios such as 0.001% and 0.0001%, our numerical results show low prediction abilities of the proposed models due to the heavy information loss caused by the aggressive sampling strategies. Figure 5 reflects the changes of the accuracy over the decreasing sampling ratios, i.e., 100%, 50%, 1%, 0.1%, 0.01%. We also performed the prediction process based on 0.001% sampling data, but we could not obtain acceptable prediction results due to many zeros occurring in the sampling dataset.

To investigate the short-term effects of intervention measures on decreasing flu epidemics, we further predicted the flu tweets volumes in the near future with flu-reduction measures. Specifically, based on each group of model parameters obtained in the above prediction process, we retained them unchanged in Equation (1) except doubling the value of c (the rate of protection), which means that, with other things being equal, humans took twice as much effort as before to reduce the incidence of influenza.

**Figure 6:**
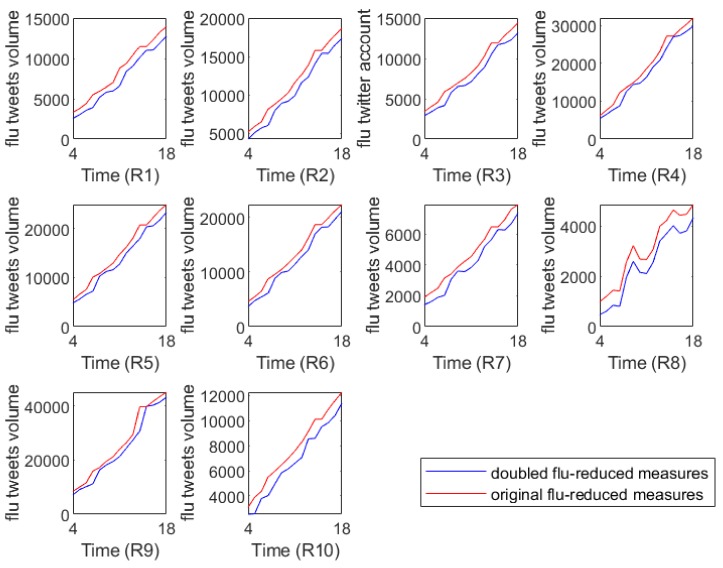
The predicted flu tweet volumes with different strengths of flu interventions. *x*-axis represents the predicted time period of 15 weeks from the 43rd week of 2018 to the 5th week of 2019, which are marked by 4–18 in the *x*-axis. The predicted flu tweets volume (flu tweet count) of each CDC region from Region 1 (marked as R1) to Region 10 (marked as R10) is shown in each subfigure.

Figure 6 illustrates the full flu tweets volumes with different flu interventions, where the red lines represent the predicted flu tweet volumes from the 43rd week of 2018 to the 5th week of 2019 and they can serve as baseline references. The blue lines capture the predicted flu tweet volumes under the double protection rate. Figure 6 shows that if stricter control measures are taken, the flu tweet volumes will be controlled in some extent. In other words, our analysis confirms the positive short-term effects of intervention measures on the spreading of flu epidemics.

## 5. Conclusions

In this paper, we developed a specific prediction model for influenza prediction based on real-time tweets data from social media. The model ensures real-time prediction and is applicable to sampling data. Our current study is specific to flu tweets, and we believe this systematic methodology can be easily extended and adapted to other types of datasets.

Our numerical simulations demonstrate that the PDE model has a robust prediction ability in the scenarios of random sampling. Our developed model achieves a higher prediction accuracy with the relative accuracy of over 90% with 1% sampling data. Furthermore, less historical data is required for prediction in our new model. Because of different datasets, it may be not comparable with other works. Nevertheless, in [2] Wang et al. achieved an accuracy of approximately 84% using the first 11-week data as a training set to predict the flu tweet count of week 12 in 2014. Broniatowski et al. [11] detected the weekly change in direction (increasing or decreasing) of influenza prevalence with 85% accuracy by analyzing the 2012–2013 influenza epidemic. Chen et al. [13] applied two weakly supervised temporal topic models to make flu case-count predictions in some countries of South America and compared their results with other results in different statistical measures.

We also incorporate the effects of the human active interventions in our mechanistic PDE model. Therefore, the effects of flu-reduction/protection intervention can be quantified. This could potentially provide some insights for the government agencies and healthcare industry to develop effective prevention strategies or educational campaigns for flu prevention.

Our future work will focus on the validations of the PDE-based prediction model on different sampling strategies such as data from community sampling and strata sampling. We are also interested in exploring the impact of tweet data sampling on the overall data quality and on the efficiency of social media applications. For instance, sampling often alters the underlying tweet distribution, and thus may reduce the efficiency of some critical social media applications such as spammer detection and sentiment modeling. We are currently addressing these problems via integrating PDE-mechanical models and data mining techniques.

## Figures and Tables

**Table 1:** The average relative accuracy of the 10 CDC regions (marked R1–R10 in the table) with various sampling ratios during our data collection period.

	R1	R2	R3	R4	R5	R6	R7	R8	R9	R10
1% Sampling	93%	93%	93%	92%	92%	92%	95%	89%	91%	94%
0.1% Sampling	91%	92%	88%	91%	93%	90%	87%	76%	90%	87%
0.01% Sampling	93%	90%	93%	88%	90%	91%	93%	75%	90%	93%

## Supplementary Materials

The following are available online at https://www.mdpi.com/1660-4601/17/3/678/s1. Table S1: The full flu tweets volumes of the 10 CDC-regions (Region 1-Region 10) from the 40th week of 2018 (2018W40) and ending on the 5th week of 2019 (2019W5).
