# Supplementary material for: Regional Influenza Prediction with Sampling Twitter Data and PDE Model
Source: Int J Environ Res Public Health. 2020 Jan 21;17(3):678. doi: 10.3390/ijerph17030678 (PMC7037800; doi:10.3390/ijerph17030678)
Supplement: Supplementary file 1 [file ijerph-17-00678-s001.pdf]

Table S1: The full flu tweets volumes of the 10 CDC-regions (Region 1-Region 10) from the 40th week of 2018 (2018W40) and ending on the 5th week of 2019 (2019W5).

Region / Week	1	2	3	4	5	6	7	8	9	10
2018W40	1652	2002	959	1365	1505	1155	427	259	2261	644
2018W41	2135	3052	1715	2744	2835	2065	980	490	3731	1554
2018W42	2695	4081	2646	4543	4221	3290	1491	749	6174	2226
2018W43	3311	5159	3395	6132	5446	4592	1897	959	8442	3094
2018W44	3780	5908	4011	7644	6622	5474	2205	1176	10031	3906
2018W45	4410	6496	4578	9051	7574	6503	2499	1337	11606	4368
2018W46	5502	8134	5880	12341	10080	8729	3143	1869	15848	5502
2018W47	5936	8792	6377	13636	10780	9555	3409	2079	17213	5978
2018W48	6454	9506	6937	14875	11872	10458	3864	2338	19530	6475
2018W49	7000	10276	7497	16422	12936	11564	4221	2590	21168	6993
2018W50	8799	11599	8239	18641	14791	12796	4557	2814	24059	7588
2018W51	9373	12635	9086	20601	16184	14028	5138	3080	26306	8260
2018W52	10451	13895	10346	23163	18046	16226	5670	3402	29400	9142
2019W1	11473	15785	11914	27251	20741	18690	6468	3843	39676	10108
2019W2	11473	15785	11914	27251	20741	18690	6468	3843	39676	10108
2019W3	12299	16849	12852	28966	22225	19894	6937	4130	41622	10885
2019W4	13265	17801	13580	30422	23688	21196	7609	4396	43407	11592
